# Neurological and Psychological Sequelae Associated With Multisystem Inflammatory Syndrome in Children

**DOI:** 10.1001/jamanetworkopen.2023.24369

**Published:** 2023-07-19

**Authors:** Caitlin K. Rollins, Johanna Calderon, David Wypij, Alex M. Taylor, Tahera Sultana Davalji Kanjiker, Julia S. Rohde, Moshe Maiman, Laura D. Zambrano, Margaret M. Newhams, Susan Rodriguez, Nicholas Hart, Jennifer Worhach, Suden Kucukak, Tina Y. Poussaint, Mary Beth F. Son, Matthew L. Friedman, Shira J. Gertz, Charlotte V. Hobbs, Michele Kong, Aline B. Maddux, Jennifer L. McGuire, Paul A. Licht, Mary Allen Staat, Lael M. Yonker, Maitreyi Mazumdar, Adrienne G. Randolph, Angela P. Campbell, Jane W. Newburger

**Affiliations:** 1 Department of Neurology, Boston Children’s Hospital, Boston, Massachusetts; 2 Department of Neurology, Harvard Medical School, Boston, Massachusetts; 3 National Institute of Health and Medical Research INSERM U1046, PhyMedExp, Montpellier, France; 4 Department of Psychiatry, Boston Children’s Hospital, Boston, Massachusetts; 5 Department of Cardiology, Boston Children’s Hospital, Boston, Massachusetts; 6 Department of Biostatistics, Harvard T.H. Chan School of Public Health, Boston, Massachusetts; 7 Department of Pediatrics, Harvard Medical School, Boston, Massachusetts; 8 Department of Psychiatry, Harvard Medical School, Boston, Massachusetts; 9 COVID-19 Response Team, Centers for Disease Control and Prevention, Atlanta, Georgia; 10 Department of Anesthesiology, Critical Care, and Pain Medicine, Boston Children’s Hospital, Boston, Massachusetts; 11 Department of Radiology, Boston Children’s Hospital, Boston, Massachusetts; 12 Department of Radiology, Harvard Medical School, Boston, Massachusetts; 13 Division of Immunology, Department of Pediatrics, Boston Children’s Hospital, Boston, Massachusetts; 14 Division of Pediatric Critical Care Medicine, Department of Pediatrics, Indiana University School of Medicine and Riley Hospital for Children, Indianapolis; 15 Division of Pediatric Critical Care, Department of Pediatrics, Cooperman Barnabas Medical Center, Livingston, New Jersey; 16 Division of Infectious Diseases, Department of Pediatrics, Department of Microbiology, University of Mississippi Medical Center, Jackson; 17 Division of Pediatric Critical Care Medicine, Department of Pediatrics, University of Alabama at Birmingham, Birmingham; 18 Department of Pediatrics, Section of Critical Care Medicine, University of Colorado School of Medicine and Children’s Hospital Colorado, Aurora; 19 Division of Neurology at The Children’s Hospital of Philadelphia, Philadelphia, Pennsylvania; 20 Department of Neurology, Perelman School of Medicine at the University of Pennsylvania, Philadelphia; 21 Department of Pediatrics, Perelman School of Medicine at the University of Pennsylvania, Philadelphia; 22 Department of Pediatrics, University of Cincinnati, Division of Infectious Diseases, Cincinnati Children’s Hospital Medical Center, Cincinnati, Ohio; 23 Department of Pediatrics, Division of Pediatric Pulmonary and Mucosal Immunology and Biology Research Center, Division of Infectious Disease, Massachusetts General Hospital, Boston; 24 Department of Anaesthesia, Harvard Medical School, Boston, Massachusetts

## Abstract

**Importance:**

Acute neurological involvement occurs in some patients with multisystem inflammatory syndrome in children (MIS-C), but few data report neurological and psychological sequelae, and no investigations include direct assessments of cognitive function 6 to 12 months after discharge.

**Objective:**

To characterize neurological, psychological, and quality of life sequelae after MIS-C.

**Design, Setting, and Participants:**

This cross-sectional cohort study was conducted in the US and Canada. Participants included children with MIS-C diagnosed from November 2020 through November 2021, 6 to 12 months after hospital discharge, and their sibling or community controls, when available. Data analysis was performed from August 2022 to May 2023.

**Exposure:**

Diagnosis of MIS-C.

**Main Outcomes and Measures:**

A central study site remotely administered a onetime neurological examination and in-depth neuropsychological assessment including measures of cognition, behavior, quality of life, and daily function. Generalized estimating equations, accounting for matching, assessed for group differences.

**Results:**

Sixty-four patients with MIS-C (mean [SD] age, 11.5 [3.9] years; 20 girls [31%]) and 44 control participants (mean [SD] age, 12.6 [3.7] years; 20 girls [45%]) were enrolled. The MIS-C group exhibited abnormalities on neurological examination more frequently than controls (15 of 61 children [25%] vs 3 of 43 children [7%]; odds ratio, 4.7; 95% CI, 1.3-16.7). Although the 2 groups performed similarly on most cognitive measures, the MIS-C group scored lower on the National Institutes of Health Cognition Toolbox List Sort Working Memory Test, a measure of executive functioning (mean [SD] scores, 96.1 [14.3] vs 103.1 [10.5]). Parents reported worse psychological outcomes in cases compared with controls, particularly higher scores for depression symptoms (mean [SD] scores, 52.6 [13.1] vs 47.8 [9.4]) and somatization (mean [SD] scores, 55.5 [15.5] vs 47.0 [7.6]). Self-reported (mean [SD] scores, 79.6 [13.1] vs 85.5 [12.3]) and parent-reported (mean [SD] scores, 80.3 [15.5] vs 88.6 [13.0]) quality of life scores were also lower in cases than controls.

**Conclusions and Relevance:**

In this cohort study, compared with contemporaneous sibling or community controls, patients with MIS-C had more abnormal neurologic examinations, worse working memory scores, more somatization and depression symptoms, and lower quality of life 6 to 12 months after hospital discharge. Although these findings need to be confirmed in larger studies, enhanced monitoring may be warranted for early identification and treatment of neurological and psychological symptoms.

## Key Points

**Question:**

Do patients with multisystem inflammatory syndrome in children (MIS-C) experience neurological and psychological sequelae 6 to 12 months after hospital discharge?

**Findings:**

In this cohort study including in-depth testing of 64 participants with MIS-C and 44 sibling and community controls, significantly more patients with MIS-C than controls (25% vs 7%) had abnormal findings on neurological examinations. Patients with MIS-C also had worse working memory, more symptoms of depression and somatization, and worse quality of life than controls.

**Meaning:**

These findings suggest that neurological and psychological sequelae may occur after hospitalization for MIS-C; although the findings need to be confirmed in larger studies, enhanced monitoring may be warranted for early identification and treatment of these symptoms.

## Teaser

This cohort study examines the neurological, psychological, and quality of life sequelae after multisystem inflammatory syndrome in children.

## Introduction

Multisystem inflammatory syndrome in children (MIS-C) is a severe complication of SARS-CoV-2 infection, with more than 9400 cases confirmed by the Centers for Disease Control and Prevention (CDC) as of May 1, 2023.^1^ This condition is characterized by fever, inflammation, multisystem organ involvement, and severe illness requiring hospitalization.^2^ Both acute COVID-19 and MIS-C may be associated with neurological signs and symptoms.^3,4,5,6^ Although estimates vary, 1 large public health registry from 2020 to 2021 reported neurologic involvement (eg, seizures, encephalopathy, neuroinflammatory changes, and corpus callosum lesions) in approximately 1 in 5 cases of MIS-C, with life-threatening neurologic involvement in less than 5% of cases.^3,4^

Few data address long-term noncardiac outcomes for MIS-C, and follow-up care varies among institutions.^7,8,9^ One study^8^ of MIS-C in the United Kingdom found that among 46 patients assessed 6 months after discharge, 39% had abnormal neurological examination, and 22% reported severe emotional difficulties on the Pediatric Quality of Life Inventory (PedsQL). Follow-up was clinic based, without a control sample for comparison, and the study was conducted amid early pandemic restrictions, which may have influenced outcomes. Recently, a clinic-based study^9^ conducted in the Netherlands found psychological and quality of life sequelae 4 months after MIS-C. Longer-term follow-up and controls were not available for comparison.

In this study, we compared patients with MIS-C 6 to 12 months after discharge vs sibling or community controls with respect to neurological and psychological outcomes, including surveys of quality of life and daily functioning. We hypothesized that patients with MIS-C would exhibit more neurological abnormalities, executive functioning difficulties, behavioral symptoms, and lower quality of life.

## Methods

### Participants

For this cohort study, we recruited patients with MIS-C and a comparison group of sibling (or if unavailable, community) controls in the US and Canada using hospital-based public health tracking lists at 9 participating institutions in the Overcoming COVID-19 network^10^ and clinic-based or online advertisements. Letters and flyers were mailed or emailed to potential participants hospitalized for MIS-C between November 2020 and November 2021, and the study team followed-up via telephone or email at 3 sites. The study was approved by the central Boston Children’s Hospital institutional review board (IRB) and was reviewed by IRBs of participating sites with CDC IRB reliance. Parents and adult participants provided verbal informed consent, and children provided verbal assent. We followed the Strengthening the Reporting of Observational Studies in Epidemiology (STROBE) reporting guideline.^11^

Cases met clinical criteria for the 2020 CDC MIS-C case definition^2^ with laboratory evidence of current or recent SARS-CoV-2 infection. Additional eligibility criteria were age 5 to 20 years at hospital discharge and age 6 years or older at evaluation to facilitate remote participation; fluency in English, Spanish, or French; access to secure wireless connection; and no hearing or vision impairment or intellectual disability precluding remote evaluation. Each case was asked to identify 1 sibling control who did not develop MIS-C; if a sibling was unavailable, cases were asked to identify a relative or friend from their community. To approximately match the demographic, geographic, and SARS-CoV-2 exposure status of cases, a control selection hierarchy was followed (eFigure 1 in Supplement 1). Controls met the same eligibility criteria as cases. Cases unable to identify a control were retained in the series.

### Demographic and Medical Information

Sociodemographic information, baseline health status, and new diagnoses after MIS-C were obtained via structured interview. For patients in the US, the Social Vulnerability Index (SVI) described social vulnerability using US Census tract data (range, 0-1 with a higher index denoting higher vulnerability).^12^ We collected data on self-reported race and ethnicity (Black or African American, multiracial, White, unknown, or other [specified by participants as Asian, Brazilian, and Middle Eastern]), as well as SVI, to evaluate potential sociodemographic-related disparities in outcomes. Variables extracted from hospital records included hospital and intensive care unit (ICU) length of stay, echocardiographic left ventricular ejection fraction (LVEF), neurological involvement, cardiopulmonary resuscitation (CPR) or shock requiring vasopressor support, extracorporeal membranous oxygenation (ECMO), inpatient corticosteroid use, neurological status at discharge examination (dichotomized as baseline vs not), and readmission within 30 days.^13^

### Neurological and Neuropsychological Assessment

We provided each participant a Wi-Fi–enabled tablet (10.2-inch iPad, eighth or ninth edition; Apple). A central team conducted assessments remotely in accordance with practice standards. The study team provided recommendations to minimize distractions during the evaluation, the examiner documented distractions that might impact validity, and scores deemed invalid were excluded. Examiners (C.K.R., J.C., A.M.T., and M.M.) were experienced in remote assessment and were blinded to group status. The duration was approximately 2 hours.

#### Neurological Examination

A board-certified pediatric neurologist (C.K.R. and 2 nonauthor individuals) conducted a standardized neurological examination according to Child Neurology Society remote guidance,^14^ including mental status, cranial nerves (excluding smell, pupillary reactions, and facial sensation), motor, coordination, and gait. Given the limitations of remote evaluation, the examination’s findings were dichotomized as normal vs abnormal on the basis of overall clinical impression.

#### Neuropsychological Assessment

A pediatric neuropsychologist (J.C., A.M.T., and M.M.) assessed cognitive function using standardized measures validated for remote administration and parent-child surveys (Table 1). Standard translated measures were used; for those that were not available, the native Spanish-speaking neuropsychologist (J.C.) translated relevant items from English to Spanish.

**Table 1.** Description of Assessment Measures

Measure	Domain	Age range	Normative score, mean (SD)	Interpretation
Wechsler Intelligence Scales (WASI-II, WISC-V, WAIS-IV)				
WASI-II FSIQ-2	Intelligence quotient estimate	All ages	100 (15)	Higher scores are better
Working Memory Index	Working memory	WISC-V, 6-15 y; WAIS-IV, 16-22 y		
Processing Speed Index	Processing speed			
National Institutes of Health Cognition Toolbox				
List Sort Working Memory	Working memory	All ages	100 (15)	Higher scores are better
Picture Sequence	Episodic memory			
Delis-Kaplan Executive Function System				
Verbal Fluency Switching	Complex inhibitory control and cognitive flexibility	8-22 y	10 (3)	Higher scores are better
Color Word Interference Switching				
BRIEF-2 or BRIEF-A				
Global Executive Composite	Daily life executive functioning	BRIEF-2, <18 y; BRIEF-A, 18-22 y	50 (10)	Lower scores are better
Behavior Assessment Scale for Children, Third Edition				
Internalizing problems	Anxiety, depression, somatization	All ages	50 (10)	Lower scores are better (60-69, at-risk, requiring monitoring; ≥70, clinically significant)
Externalizing problems	Hyperactivity, aggression, conduct problems			
Behavioral Symptoms Index	Hyperactivity, aggression, depression, atypicality, withdrawal, attention problems			
Adaptive Skills Composite	Adaptive function			Higher scores are better
PedsQL				
PedsQL4.0 Generic	Health-related quality of life	6-22 y	Range 0-100	Higher scores are better
PedsQL Multidimensional Fatigue Scale	General, sleep-rest and cognitive fatigue			
Patient-Reported Outcomes Measurement Information System				
Sleep disturbance	Perceptions of sleep quality, sleep depth, and restoration associated with sleep	Self-report, ≥8 y; parent proxy, 5-7 y	50 (10)	Lower scores are better
Sleep impairment	Perceptions of alertness, sleepiness, and tiredness during usual waking hours			
Functional Disability Inventory, total score	Challenges in daily activity	Self-report, ≥8 y; parent proxy, 5-7 y	Range 0-60	Lower scores are better

Abbreviations: BRIEF-2, Behavior Rating Inventory of Executive Function, Version 2; BRIEF-A, Behavior Rating Inventory of Executive Function, Adult Edition; FSIQ-2, Full-Scale Intelligence Quotient–2 subtest version; PedsQL, Pediatric Quality of Life Inventory; WAIS-IV, Wechsler Adult Intelligence Scale, Fourth Edition; WASI-II, Wechsler Abbreviated Scale of Intelligence, Second Edition; WISC-V, Wechsler Intelligence Scale for Children, Fifth Edition.

To test general cognitive ability, the 2-subtest version of the Wechsler Abbreviated Scale of Intelligence, Second Edition,^15^ generated a time-efficient and reliable estimate of general cognitive ability. The primary measure of working memory was the National Institutes of Health (NIH) Cognition Toolbox List Sort Working Memory Test^16^ supplemented by the Working Memory Index of the Wechsler Intelligence Scale for Children, Fifth Edition,^17^ or the Wechsler Adult Intelligence Scale–IV.^18^ Children completed 2 subtests of the Delis-Kaplan Executive Function System^19^ to measure inhibitory control and cognitive flexibility. Analysis was confined to Verbal Fluency Switching and Color-Word Interference Switching scores, because these higher-order executive processes may be most sensitive to group differences. The Behavioral Rating Inventory of Executive Function, Second Edition (BRIEF-2)^20^ or adult version (BRIEF-A)^21^ described daily life executive functioning.

The Processing Speed Index of Wechsler Intelligence Scale for Children, Fifth Edition, or the Wechsler Adult Intelligence Scale–IV measured processing speed. The NIH Cognition Toolbox Picture Sequence Memory Test^16^ evaluated episodic memory (the ability to encode and retrieve specific events and information). The Behavior Assessment Scale for Children Third Edition (BASC-3)^22^ parent report provided scores for psychological outcomes (eg, depression, anxiety, and somatization).

Parents and participants completed the PedsQL 4.0 Generic Core Scales^23^ and the PedsQL Multidimensional Fatigue Scale.^24^ Participants completed 4-item versions of the Patient Reported Outcomes Measurement Information System Sleep Related Impairment and Sleep Disturbance Scales (substituting parent proxy as needed).^25^ The Functional Disability Inventory^26^ described challenges performing daily activities in home, school, recreational, or social domains.

### Statistical Analysis

Data analysis was performed from August 2022 to May 2023. We conducted statistical analyses using SAS statistical software version 9.4 (SAS Institute), generally involving 95% CIs without correction for multiple comparisons, and graphical analysis with R Studio version 3 (R Project for Statistical Computing). Analyses excluded participants who were missing data for that variable. The primary comparison was MIS-C vs controls. Secondarily, we compared MIS-C vs normative data, estimated the proportion of children with MIS-C whose scores exceeded clinical thresholds, and explored demographic and medical associations with outcomes.

#### Comparison of MIS-C Group With Control Group or Normative Data

Linear regression for continuous outcomes or logistic regression for dichotomous outcomes was used to compare groups using generalized estimating equations with the exchangeable working correlation assumption and robust variances. Use of generalized estimating equations allowed the inclusion of MIS-C cases without a matched control in the analyses by accounting for clustering within matched pairs. A sensitivity analysis was performed including only matched pairs. We also conducted a sensitivity analysis to assess whether substantial preexisting neuropsychiatric conditions might affect the findings, excluding participants with baseline attention-deficit/hyperactivity disorder (ADHD) unmedicated at assessment, anxiety disorder, or autism spectrum disorder (ASD).

#### Within MIS-C Group Comparison With Thresholds and Demographic and Medical Variables

For selected outcomes reflecting broad domains of functioning, we compared the percentage of children with MIS-C who met clinical thresholds with exact binomial 95% CIs to normative expectations (ie, 16%). We defined our clinical threshold as 1 SD below the mean, consistent with relevant test manuals (eg, BASC-3^22^ and BRIEF^20,21^) or published guidelines.^27^ Threshold analysis was considered secondary because of uncertainty regarding the applicability of historical data during a pandemic. Within the MIS-C group, univariable linear regression explored whether demographic or hospitalization-related variables were associated with selected outcome measures that capture broad domains of functioning or showed group differences in the primary analysis.

## Results

Families of 250 eligible children were sent recruitment flyers; the study team followed up with 78 potential participants via phone or email. In addition, 13 individuals from the US and Canada responded to online or clinic-based advertisements. Sixty-eight cases and 48 controls enrolled. Two pairs never completed evaluations, and 2 pairs recruited via advertisement were later excluded because of medical record review suggesting an alternative diagnosis, yielding 64 patients with MIS-C (mean [SD] age, 11.5 [3.9] years; 20 girls [31%]) and 44 control participants (mean [SD] age, 12.6 [3.7] years; 20 girls [45%]) who were included in analyses (eFigure 2 in Supplement 1). Among controls approached, 96% participated, with nonparticipants reporting inadequate time or interest. Most controls were siblings of cases (39 controls), including 3 twins (1 identical and 2 fraternal) and 4 half-siblings; 2 relatives and 3 friends participated. No French speakers enrolled; 1 case and 1 control were bilingual in Spanish, but all child assessments were conducted in English.

The matched case and control groups were similar with respect to sex, race, ethnicity, social vulnerability,^12^ and primary caregiver education, although matched cases were slightly younger than their controls (−0.9 years; 95% CI, −1.6 to −0.2 years). The unmatched cases were similar to matched cases on all demographic and baseline health variables (Table 2). The sample was predominantly White, privately insured, and of low social vulnerability. Although fewer cases had baseline medical or neurobehavioral conditions than controls, these differences did not reach statistical significance. When asked whether they had received a COVID-19 vaccine, 2 of 62 cases and 3 of 37 controls reported receiving 2 doses before the case MIS-C illness.

**Table 2.** Demographic and Medical Characteristics of Participants by Group

Characteristic^a^	Participants, No. (%)	
	Patients with MIS-C (n = 64)	Control group (n = 44)
Age, mean (SD), y	11.5 (3.9)	12.6 (3.7)
Sex		
Female	20 (31)	20 (45)
Male	44 (69)	24 (55)
Race		
Black or African American	12 (19)	6 (14)
Multiracial	6 (9)	4 (9)
White	40 (63)	28 (64)
Other or unknown^b^	6 (9)	6 (14)
Hispanic ethnicity	10 (16)	5 (11)
Social Vulnerability Index, mean (SD)	0.39 (0.26)	0.40 (0.28)
Primary caregiver education		
Graduated college or greater	38 (59)	27 (61)
Some college	18 (28)	10 (23)
Grade 12 or less	8 (13)	6 (14)
No primary caregiver	0	1 (2)
Private insurance	46 (73)	35 (83)
Single family home	54 (84)	39 (89)
Baseline medical conditions	19 (30)	7 (17)
Neurological^c^	1 (2)	1 (2)
Respiratory^d^	7 (11)	4 (10)
Rheumatologic or autoimmune	1 (2)	0
Gastrointestinal or hepatic	4 (6)	0
Endocrine	5 (8)	1 (2)
Obesity	7 (11)	4 (10)
Other^e^	4 (6)	0
Baseline psychological conditions^f^	11 (17)	4 (10)

Abbreviation: MIS-C, multisystem inflammatory syndrome in children.

^a^ Data were complete except for Social Vulnerability Index (62 MIS-C cases and 41 controls), private insurance (63 MIS-C cases and 41 controls), and baseline medical and psychological conditions (63 MIS-C cases and 42 controls).

^b^ Other or unknown race refers to self-reported as Asian (3 MIS-C cases and 3 controls), Brazilian (1 MIS-C case and 1 control), Middle Eastern (1 MIS-C case and 1 control), and declined to answer (1 MIS-C case and 1 control).

^c^ Neurological conditions included dopa-responsive dystonia (1 MIS-C case) and Guillain-Barre syndrome (1 control).

^d^ Respiratory conditions included asthma (7 MIS-C cases and 3 controls) and obstructive sleep apnea (1 control).

^e^ Other conditions included osteochondritis dissecans (1 MIS-C case), horseshoe kidney (1 MIS-C case), postural orthostatic tachycardia syndrome (1 MIS-C case), and aortic valve abnormality (1 MIS-C case).

^f^ Psychological conditions included attention-deficit/hyperactivity disorder (ADHD; 4 MIS-C cases and 2 controls); anxiety (1 MIS-C case and 1 control); autism spectrum disorder (ASD; 2 MIS-C cases and 1 control); ADHD and dyslexia (1 MIS-C case); ADHD and ASD (1 MIS-C case); ADHD, ASD, and anxiety (1 MIS-C case); and anxiety and posttraumatic stress disorder (1 MIS-C case).

Among patients with MIS-C, the median (IQR) length of hospital stay was 5 (4-7) days, with 35 of 64 cases (55%) receiving ICU care and 17 of 62 cases (27%) with severe hemodynamic compromise (14 with shock requiring vasopressors, 6 receiving CPR, and 1 undergoing ECMO). LVEF was depressed in 24 of 62 cases (39%), 3 severely (LVEF <35%). Neurologic involvement was present in 13 of 62 cases (21%) during their clinical course. Steroids were administered to 54 of 63 cases (86%) while inpatient, and 5 of 63 patients (8%) were readmitted within 30 days (4 with ongoing MIS-C symptoms, and 1 with orthostatic hypotension). The median (IQR) duration between discharge and evaluation was 7.9 (6.1-11.0) months.

### Neurological and Neuropsychological Assessment

#### Comparison of MIS-C With Control Group and Normative Data

Neurological examination findings at evaluation were abnormal more often in the patients with MIS-C (15 of 61 children [25%]) than in the control group (3 of 43 children [7%]; odds ratio, 4.7; 95% CI 1.3-16.7). Motor, coordination, and gait abnormalities were most common (eTable 1 in Supplement 1). The groups had comparable scores on most cognitive assessments; however, the MIS-C group scored lower than the control group on the NIH List Sort Working Memory Test (mean [SD] score, 96.1 [14.3] vs 103.1 [10.5]) (Table 3 and Figure). The MIS-C group had more internalizing symptoms on the BASC-3 (mean [SD] score, 53.4 [13.8] vs 47.8 [8.1]), with higher scores vs the control group for somatization (mean [SD] score, 55.5 [15.5] vs 47.0 [7.6]) and depression (mean [SD] score, 52.6 [13.1] vs 47.8 [9.4]). Both self-reported (mean [SD] score, 79.6 [13.1] vs 85.5 [12.3]) and parent-reported (mean [SD] score, 80.3 [15.5] vs 88.6 [13.0]) ratings indicated lower quality of life, particularly psychosocial health, in the MIS-C group vs the control group. Sleep impairment and disturbance did not differ between groups, but fatigue was more common among the MIS-C group, especially in the sleep-rest domain. New formal diagnosis of ADHD, anxiety, or depression after hospitalization was more common in the MIS-C than control group (8 of 63 children vs 1 of 41 children; odds ratio, 4.5; 95% CI, 1.1-18.1; ADHD, 3 cases and 0 controls; anxiety, 6 cases and 0 controls; depression, 3 cases and 1 control); some children developed multiple conditions. Adjustments for sex in models comparing outcomes between groups did not appreciably alter the findings. The pattern of results comparing with population norms (Table 3) and sensitivity analyses including only matched pairs (eTable 2 in Supplement 1) and removing 13 participants (11 cases and 2 controls) with a preexisting diagnosis of ADHD (unmedicated), anxiety disorder, or ASD (eTable 3 in Supplement 1) attenuated some group differences but yielded an overall pattern of findings similar to that for the primary analysis.

**Table 3.** Psychological Outcomes by Group and Comparison of MIS-C Cases vs Controls and vs Population Norms

Outcome^a^	Score, mean (SD)		MIS-C cases vs controls		MIS-C cases vs population norms	
	MIS-C cases (n = 64)	Controls (n = 44)	Mean difference (95% CI)^b^	SMD (95% CI)^b^^,^^c^	Mean difference (95% CI)	SMD (95% CI)^c^
Wechsler Intelligence Scales (WASI-II, WISC-V, WAIS-IV)						
Intelligence quotient estimate	100.9 (14.6)	103.7 (14.1)	−2.3 (−6.6 to 1.9)	−0.15 (−0.44 to 0.12)	0.9 (−2.6 to 4.5)	0.06 (−0.17 to 0.30)
Working Memory Index	99.1 (15.0)	103.0 (15.5)	−3.7 (−8.9 to 1.5)	−0.24 (−0.59 to 0.10)	−0.9 (−4.6 to 2.7)	−0.06 (−0.31 to 0.18)
Processing Speed Index	97.7 (15.5)	96.8 (14.3)	0.3 (−5.5 to 6.1)	0.02 (−0.37 to 0.41)	−2.3 (−6.7 to 2.2)	−0.15 (−0.45 to 0.15)
National Institutes of Health Cognition Toolbox						
List Sort Working Memory	96.1 (14.3)	103.1 (10.5)	−6.9 (−11.6 to −2.2)	−0.46 (−0.77 to −0.15)	−3.9 (−7.4 to −0.3)	−0.26 (−0.49 to −0.02)
Picture Sequence Memory	99.0 (15.1)	102.8 (18.0)	−2.8 (−8.4 to 2.8)	−0.19 (−0.56 to 0.19)	−1.0 (−4.6 to 2.7)	−0.07 (−0.31 to 0.18)
Delis-Kaplan Executive Function System						
Verbal Fluency Switching	10.7 (3.1)	10.7 (2.6)	0.0 (−1.2 to 1.2)	0.00 (−0.40 to 0.40)	0.7 (−0.1 to 1.5)	0.23 (−0.03 to 0.50)
Color-Word Interference Switching	9.0 (3.2)	9.7 (3.2)	−0.7 (−1.9 to 0.5)	−0.23 (−0.63 to 0.17)	−1.0 (−1.9 to −0.1)	−0.33 (−0.63 to –0.03)
BRIEF Global Executive Composite^d^	52.6 (11.5)	50.7 (10.7)	1.3 (−2.0 to 4.6)	0.13 (−0.20 to 0.46)	2.6 (−0.2 to 5.4)	0.26 (−0.02 to 0.54)
Behavior Assessment Scale for Children, Third Edition						
Internalizing Problems	53.4 (13.8)	47.8 (8.1)	4.9 (1.4 to 8.4)	0.49 (0.14 to 0.84)	3.4 (−0.1 to 6.9)	0.34 (−0.01 to 0.69)
Anxiety	50.4 (11.3)	49.7 (10.4)	0.3 (−3.4 to 4.0)	0.03 (−0.34 to 0.40)	0.4 (−2.4 to 3.3)	0.04 (−0.24 to 0.33)
Depression	52.6 (13.1)	47.8 (9.4)	4.0 (0.4 to 7.6)	0.40 (0.04 to 0.76)	2.6 (−0.8 to 5.9)	0.26 (−0.08 to 0.59)
Somatization	55.5 (15.5)	47.0 (7.6)	8.1 (4.2 to 12.0)	0.81 (0.42 to 1.20)	5.5 (1.5 to 9.4)	0.55 (0.15 to 0.94)
Externalizing problems	50.0 (11.0)	47.1 (8.2)	2.6 (−0.5 to 5.7)	0.26 (−0.05 to 0.57)	0.0 (−2.8 to 2.8)	0.00 (−0.28 to 0.28)
Hyperactivity	50.6 (12.0)	47.3 (9.7)	3.2 (−0.8 to 7.1)	0.32 (−0.08 to 0.71)	0.6 (−2.4 to 3.7)	0.06 (−0.24 to 0.37)
Aggression	50.4 (11.9)	48.0 (7.0)	2.1 (−1.0 to 5.1)	0.21 (−0.10 to 0.51)	0.4 (−2.6 to 3.4)	0.04 (−0.26 to 0.34)
Conduct problems	48.9 (8.2)	46.8 (7.7)	2.0 (−0.4 to 4.5)	0.20 (−0.04 to 0.45)	−1.1 (−3.2 to 1.0)	−0.11 (−0.32 to 0.10)
Behavioral Symptoms Index^e^	50.6 (12.3)	47.3 (10.0)	2.6 (−0.8 to 6.1)	0.26 (−0.08 to 0.61)	0.6 (−2.5 to 3.8)	0.06 (−0.25 to 0.38)
Atypicality	49.3 (10.9)	48.0 (10.9)	1.0 (−2.9 to 4.9)	0.10 (−0.29 to 0.49)	−0.7 (−3.4 to 2.1)	−0.07 (−0.34 to 0.21)
Withdrawal	50.1 (12.0)	48.3 (10.4)	1.5 (−2.5 to 5.5)	0.15 (−0.25 to 0.55)	0.1 (−3.0 to 3.1)	0.01 (−0.30 to 0.31)
Attention problems	49.7 (11.9)	47.5 (10.1)	1.9 (−1.8 to 5.6)	0.19 (−0.18 to 0.56)	−0.3 (−3.3 to 2.7)	−0.03 (−0.33 to 0.27)
Adaptive skills composite	51.5 (11.1)	52.6 (11.3)	−0.8 (−3.8 to 2.3)	−0.08 (−0.38 to 0.23)	1.5 (−1.3 to 4.3)	0.15 (−0.13 to 0.43)
PedsQL Generic						
Self-report total	79.6 (13.1)	85.5 (12.3)	−4.7 (−8.9 to 0.5)	−0.36 (−0.68 to 0.04)	−3.3 (−6.7 to 0.1)	−0.25 (−0.51 to 0.01)
Physical health	85.2 (15.0)	89.6 (11.9)	−3.6 (−7.9 to 0.8)	−0.26 (−0.57 to 0.06)	−1.7 (−5.6 to 2.2)	−0.12 (−0.40 to 0.16)
Psychosocial health	76.6 (14.5)	83.3 (14.4)	−5.4 (−10.3 to −0.5)	−0.37 (−0.70 to −0.03)	−4.1 (−7.9 to −0.3)	−0.28 (−0.54 to −0.02)
Parent-report total	80.3 (15.5)	88.6 (13.0)	−6.9 (−11.4 to −2.4)	−0.43 (−0.72 to −0.15)	−1.1 (−5.0 to 2.9)	−0.07 (−0.31 to 0.18)
Physical health	86.9 (15.6)	91.0 (18.5)	−3.3 (−9.8 to 3.2)	−0.17 (−0.49 to 0.16)	3.6 (−0.4 to 7.6)	0.18 (−0.02 to 0.38)
Psychosocial health	76.8 (17.5)	87.4 (12.3)	−8.8 (−12.9 to −4.7)	−0.55 (−0.81 to −0.30)	−3.4 (−7.9 to 1.0)	−0.22 (−0.50 to 0.06)
PedsQL Multidimensional Fatigue						
Self-report total	74.9 (17.9)	81.6 (14.0)	−5.0 (−9.4 to −0.7)	−0.38 (−0.71 to −0.05)	−5.6 (−10.2 to −0.9)	−0.42 (−0.77 to 0.07)
General fatigue	79.5 (18.4)	85.0 (18.0)	−4.6 (−11.2 to 2.0)	−0.31 (−0.75 to 0.13)	−5.8 (−10.6 to −1.0)	−0.39 (−0.71 to −0.07)
Sleep-rest fatigue	72.4 (21.3)	79.6 (17.1)	−6.9 (−12.5 to −1.2)	−0.37 (−0.67 to −0.06)	−2.6 (−8.1 to 2.9)	−0.14 (−0.43 to 0.15)
Cognitive fatigue	72.8 (23.7)	80.2 (17.7)	−5.8 (−12.3 to 0.6)	−0.33 (−0.71 to 0.03)	−8.3 (−14.4 to −2.1)	−0.48 (−0.83 to −0.12)
Parent-report total	78.8 (20.8)	87.6 (17.0)	−7.6 (−13.9 to −1.3)	−0.67 (−1.22 to −0.11)	−10.9 (−16.2 to −5.5)	−0.96 (−1.42 to −0.48)
General fatigue	79.8 (22.3)	88.4 (18.7)	−7.3 (−14.4 to −0.2)	−0.55 (−1.08 to −0.02)	−9.5 (−15.2 to −3.8)	−0.71 (−1.14 to −0.29)
Sleep-rest fatigue	78.9 (22.6)	88.5 (16.3)	−9.4 (−16.5 to −2.3)	−0.64 (−1.12 to −0.16)	−10.0 (−15.7 to −4.2)	−0.68 (−1.07 to −0.29)
Cognitive fatigue	77.7 (26.2)	86.1 (24.0)	−6.8 (−15.5 to 1.9)	−0.45 (−1.02 to 0.13)	−13.1 (−19.7 to −6.3)	−0.86 (−1.30 to −0.41)
PROMIS Sleep Disturbance	53.8 (9.5)	50.8 (7.7)	3.0 (−0.4 to 6.3)	0.30 (−0.04 to 0.63)	3.8 (1.4 to 6.1)	0.38 (0.14 to 0.61)
PROMIS Sleep Impairment	48.3 (9.4)	47.5 (9.3)	0.5 (−2.3 to 3.2)	0.05 (−0.23 to 0.32)	−1.7 (−4.1 to 0.6)	−0.17 (−0.41 to 0.06)
Functional Disability Inventory	4.1 (5.7)	2.6 (3.9)	1.4 (−0.4 to 3.3)	0.36 (−0.10 to 0.85)	NA	NA

Abbreviations: BRIEF, Behavior Rating Inventory of Executive Function; MIS-C, multisystem inflammatory syndrome in children; NA, not applicable; PedsQL, Pediatric Quality of Life Inventory; PROMIS, Patient Reported Outcomes Measurement Information System; SMD, standardized mean difference; WAIS-IV, Wechsler Adult Intelligence Scale, Fourth Edition; WASI-II, Wechsler Abbreviated Scale of Intelligence, Second Edition; WISC-V, Wechsler Intelligence Scale for Children, Fifth Edition.

^a^ Numbers of participants completing each assessment are as follows: Intelligence quotient estimate, 63 MIS-C cases and 42 controls; Working Memory Index, 64 MIS-C cases and 41 controls; Processing Speed Index, 46 MIS-C cases and 33 controls (resulting from nonreturn of mailed booklets); List Sort Working Memory, 62 MIS-C cases and 41 controls; Picture Sequence Memory, 64 MIS-C cases and 42 controls; Delis-Kaplan Executive Function System, 52 of 54 MIS-C cases and 38 of 40 controls (given to participants aged ≥8 years); BRIEF, 63 MIS-C cases and 43 controls; Behavior Assessment Scale for Children, Third Edition, 58 MIS-C cases and 40 controls; PedsQL self-report, 56 MIS-C cases and 39 controls; PedsQL parent-report, 58 MIS-C cases and 38 controls (due to survey noncompletion); PROMIS, 60 MIS-C cases and 39 controls; and Functional Disability Inventory, 56 MIS-C cases and 40 controls.

^b^ Adjusted for matched pairs.

^c^ SMD is the effect size relative to 1 SD based on test parameters or normative data except for Functional Disability Inventory where the control sample SD was substituted because of a lack of well-established normative data.

^d^ Includes BRIEF-2 and BRIEF-A.

^e^ Includes depression, hyperactivity, aggression, atypicality, withdrawal, attention.

**Figure.**
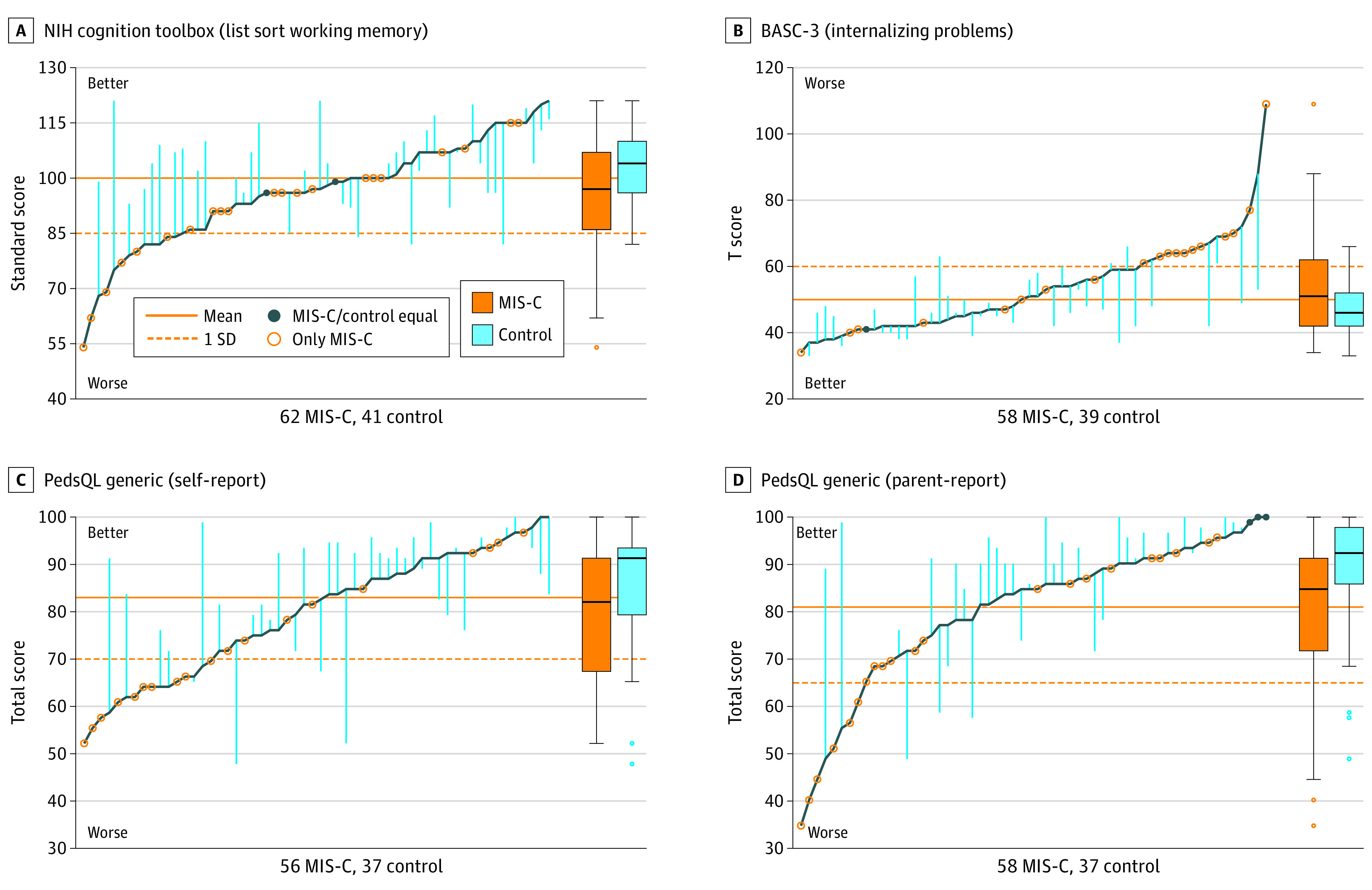
Parallel Line Plot Depicting Group Differences in Selected Outcome Measures Scores on the National Institutes of Health (NIH) Cognition Toolbox (List Sort Working Memory) (A), Behavior Assessment Scale for Children, Third Edition (BASC-3; Internalizing Problems) (B), Pediatric Quality of Life Inventory (PedsQL) Generic (Self-report) (C), and PedsQL Generic (Parent-report) (D) for patients with multisystem inflammatory syndrome in children (MIS-C) are plotted along the dark blue line in sequential order. Vertical light blue lines reflect difference between case and matched control, where available. Solid orange line reflects test mean or historical normative mean if not standardized. Dotted orange line indicates 1 SD worse than the mean; for BASC-3 internalizing problems, the dotted line indicates the test defined at-risk threshold. In the box plots, lines within boxes denote medians, ends of boxes denote IQRs, error bars extend to the farthest point within 1.5 IQR of the quartiles, and circles denote outliers.

#### Within MIS-C Group Comparison With Thresholds and Within MIS-C Group Outcome Associations With Demographic and Medical Data

The proportion of participants exceeding clinical thresholds was elevated for the BASC-3 Internalizing Composite, the BRIEF Global Executive Composite, and the PedsQL self-report (Table 4). Sixteen of 58 children (30%) had an internalizing composite score of 60 or greater. Higher social vulnerability, lower LVEF, and ICU admission were associated with worse executive functioning (List Sort Working Memory, −2.5 points per 0.1 change in SVI [95% CI, −3.8 to −1.1 points]; Color-Word Interference Switching, −1.9 points for ICU admission [95% CI, −3.6 to −0.3 points] and −1.4 points per LVEF category [95% CI, −2.3 to −0.4 points]) (eTable 4 in Supplement 1). Abnormal neurological examination findings at follow-up evaluation were not associated with psychological outcomes.

**Table 4.** Patients With Multisystem Inflammatory Syndrome in Children Meeting Clinical Threshold of >1 SD Worse Than Population Mean

Outcome (1 SD threshold)	Participants, No./total No.	Percentage (exact 95% CI)
National Institutes of Health Toolbox List Sort Working Memory Test (≤85)	14/62	23 (13-35)
D-KEFS Verbal Fluency Switching (≤7)	8/52	15 (70-28)
D-KEFS Color-Word Interference Switching (≤7)	14/52	27 (16-41)
Behavior Assessment Scale for Children, 3rd Ed		
Internalizing Composite (≥60)	16/58	30 (17-41)^a^
Externalizing Composite (≥60)	9/58	16 (7-27)
Behavioral Symptoms Index (≥60)	10/58	17 (8-29)
Adaptive Skills Composite Score (≤40)	10/58	17 (9-29)
BRIEF Global Executive Composite (≥60)^b^	18/63	29 (18-41)^a^
PedsQL Self-Report Total (≤70)	16/56	29 (17-42)^a^
PedsQL Parent-Report Total (≤65)	8/58	21 (6-25)

Abbreviations: BRIEF, Behavior Rating Inventory of Executive Function; D-KEFS, Delis-Kaplan Executive Function System; PedsQL, Pediatric Quality of Life Inventory.

^a^ Denotes outcomes for which the 95% CI is more extreme than 1 SD (for a normal distribution, 16%); thus, a higher percentage of participants meet the clinical threshold than normative data.

^b^ Includes BRIEF-2 and BRIEF-A.

## Discussion

In this cohort study, we examined neurological and psychological outcomes in children and adolescents with MIS-C, compared with a sibling and community control group. The groups had comparable scores on most cognitive assessments, but the MIS-C group exhibited more neurological abnormalities, worse working memory performance, and more behavioral symptoms, especially somatization and depression, than the control group. Quality of life, particularly psychosocial health, was lower in the MIS-C group. Fatigue was also more common among participants with MIS-C. Abnormal neurological examination findings at follow-up were not associated with adverse psychological outcomes. Collectively, the data suggest that 6 to 12 months after hospitalization for MIS-C, patients may experience neurological and psychological sequelae affecting executive functioning, internalizing symptoms, quality of life, and fatigue.

These findings extend prior work in several ways. This study includes direct assessment of cognition and parent-reported psychological health at 6 to 12 months after discharge and harnesses a contemporaneously assessed control group (largely siblings) from a similar genetic and sociodemographic background as the cases, including diverse communities. Previous studies have used historical or normative data, with unclear applicability in the present era. This sibling and community control design may distinguish adverse psychological consequences associated with the pandemic (eg, school disruption or lifestyle changes) vs those associated with MIS-C.

Two earlier clinic-based studies^8,9^ directly assessed longer-term neurological outcomes in children after MIS-C. Penner et al^8^ found that 6 months after hospitalization, 39% of children had abnormal neurological examination findings, compared with 25% in our study. Our lower rate of neurological abnormalities may reflect fewer neurological sequelae associated with more recent SARS-CoV-2 variants, earlier recognition of MIS-C, relaxation of restrictions, or improvements in MIS-C treatment.^28,29^ A recent Dutch study^9^ of children hospitalized in the ICU for MIS-C found normal intelligence but increased emotional problems and lower quality of life than population norms 4 months after hospitalization. We found a similar pattern in MIS-C cases compared with community-based controls more than 6 months after discharge, despite easing pandemic restrictions that may have affected physical activity, mental health care, and education. In our study, 30% of children with MIS-C scored in the at-risk range for everyday life executive functions and internalizing symptoms. These findings identify children who may have self-regulatory and organizational challenges or heightened risk for mood or anxiety disorders and who should undergo clinical evaluation.

Physiologic factors related to MIS-C have the potential to cause neurological insult. Severe inflammation and hematologic abnormalities may promote thrombosis or hemorrhage, hemodynamic compromise is associated with increased risk for cerebral ischemia or stroke, and steroids may have adverse behavioral effects.^30,31^ Our study found that many hospitalization-related variables (eg, length of stay, shock, CPR, ECMO, inflammation, or steroids) were not associated with neurobehavioral outcomes. However, lower LVEF and ICU admission were each significantly associated with worse executive functioning, perhaps reflecting the impact of the hyperinflammatory process on brain and heart; a direct causal effect is less likely given lack of association with other variables reflecting severe hemodynamic compromise. Larger studies are needed to confirm these exploratory analyses and their fundamental mechanisms.

Our findings can be considered in the context of other medically complex populations. Approximately one-half of the current sample required ICU admission. Post–Intensive Care Unit Syndrome in Pediatrics, a constellation of cognitive, physical, and mental health impairments after ICU admission, may reflect changes in brain function (eg, new neurologic morbidity), family dynamics, or physical changes.^32,33^ New morbidities in 1 domain (eg, cognition) may interact with other domains (eg, social reintegration) and affect psychological health.^34^ Beyond the ICU, there is no ideal comparison group with a well-defined neurobehavioral profile.

Substantial attention has focused on postacute sequelae of COVID-19, or post–COVID-19 conditions, which may include brain fog, depression, and poor sleep.^35,36,37^ The pathophysiological process remains unclear, but postulated mechanisms include neuroinflammation, deconditioning, autonomic disturbances, and posttraumatic stress. Given the substantial overlap between the psychological profile in the MIS-C cases and reported post–COVID-19 symptoms, common mechanisms may underlie the findings in these 2 diagnoses.

### Limitations

This study has limitations that should be mentioned. Children experiencing neurological or psychological symptoms (before or after MIS-C) may have been more likely to participate, creating selection bias. Neurobehavioral conditions were somewhat more common in the MIS-C group, although the sensitivity analysis showed similar findings after excluding patients with baseline ADHD (unmedicated), anxiety, or ASD. Our study design cannot distinguish hospitalization effects from those of MIS-C. The sibling control group shared a socioeconomic and school setting and SARS-CoV-2 household exposure with cases; however, we were unable to confirm COVID-19 history in controls. Baseline neurological and psychological data were not available. Our study was designed to comprehensively assess many psychological outcomes for hypothesis generation; thus, we did not control for multiple comparisons. The evaluation was conducted remotely by a central core, which limited direct assessment of some domains (eg, attention) and may have impacted performance; this effect is expected to be similar between groups. We used self-report for new diagnoses and did not obtain the number of health care encounters. Furthermore, our sample had a higher percentage of participants with private insurance, White race, and social advantage than the North American population overall, limiting generalizability. Even in this socioeconomically advantaged sample, significant reductions in psychological functioning and quality of life were noted when compared with population norms. Future larger studies should explore sequelae among diverse groups.

## Conclusions

In this cohort study of children with MIS-C and matched controls, we found that patients hospitalized for MIS-C may experience neurological and psychological sequelae 6 to 12 months after discharge. Areas of concern include working memory and daily-life executive functioning, somatization, depression, quality of life, and fatigue. The findings cannot be explained by pandemic-related environmental changes or overt neurological injury detectable on remote examination, but lower LVEF and ICU admission were associated with worse executive function in exploratory analyses. Results of the study may inform research on post–COVID-19 and other inflammatory conditions. Until larger studies validate findings and assess longer-term neurobehavioral outcomes, enhanced neurodevelopmental monitoring after hospitalization for MIS-C may be warranted for early identification and treatment of neurological and psychological symptoms.

## Notes

Supplement 1. **eFigure 1.** Control Selection Hierarchy **eFigure 2.** Enrollment Flowchart **eTable 1.** Abnormal Neurological Examination Findings by Group **eTable 2.** Sensitivity Analysis Including Matched Pairs Only **eTable 3.** Sensitivity Analysis Excluding Patients With ADHD (Unmedicated), Anxiety, and Autism **eTable 4.** Associations Between Selected Demographic and Hospitalization-Related Medical Variables and Neuropsychological Outcome Measures Among MIS-C Participants

Supplement 2.
Nonauthor Collaborators

Supplement 3.
Data Sharing Statement
