# Supplementary material for: Neurological and Psychological Sequelae Associated With Multisystem Inflammatory Syndrome in Children
Source: JAMA Netw Open. 2023 Jul 19;6(7):e2324369. doi: 10.1001/jamanetworkopen.2023.24369 (PMC10357334; doi:10.1001/jamanetworkopen.2023.24369)
Supplement: Supplement 1. — eFigure 1. Control Selection Hierarchy eFigure 2. Enrollment Flowchart eTable 1. Abnormal Neurological Examination Findings by Group eTable 2. Sensitivity Analysis Including Matched Pairs Only eTable 3. Sensitivity Analysis Excluding Patients With ADHD (Unmedicated), Anxiety, and Autism eTable 4. Associations Between Selected Demographic and Hospitalization-Related Medical Variables and Neuropsychological Outcome Measures Among MIS-C Participants [file jamanetwopen-e2324369-s001.pdf]

Supplemental Online Content

Rollins CK, Calderon J, Wypij D, et al; Overcoming COVID-19 Investigators. Neurological and psychological sequelae associated with multisystem inflammatory syndrome in children. *JAMA Netw Open*. 2023;6(7):e2324369. doi:10.1001/jamanetworkopen.2023.24369

eFigure 1. Control Selection Hierarchy
eFigure 2. Enrollment Flowchart
eTable 1. Abnormal Neurological Examination Findings by Group
eTable 2. Sensitivity Analysis Including Matched Pairs Only
eTable 3. Sensitivity Analysis Excluding Patients With ADHD (Unmedicated), Anxiety, and Autism
eTable 4. Associations Between Selected Demographic and Hospitalization-Related Medical Variables and Neuropsychological Outcome Measures Among MIS-C Participants

This supplemental material has been provided by the authors to give readers additional information about their work.

© 2023 Rollins CK et al. *JAMA Network Open*

eFigure 1.
Control Selection Hierarchy

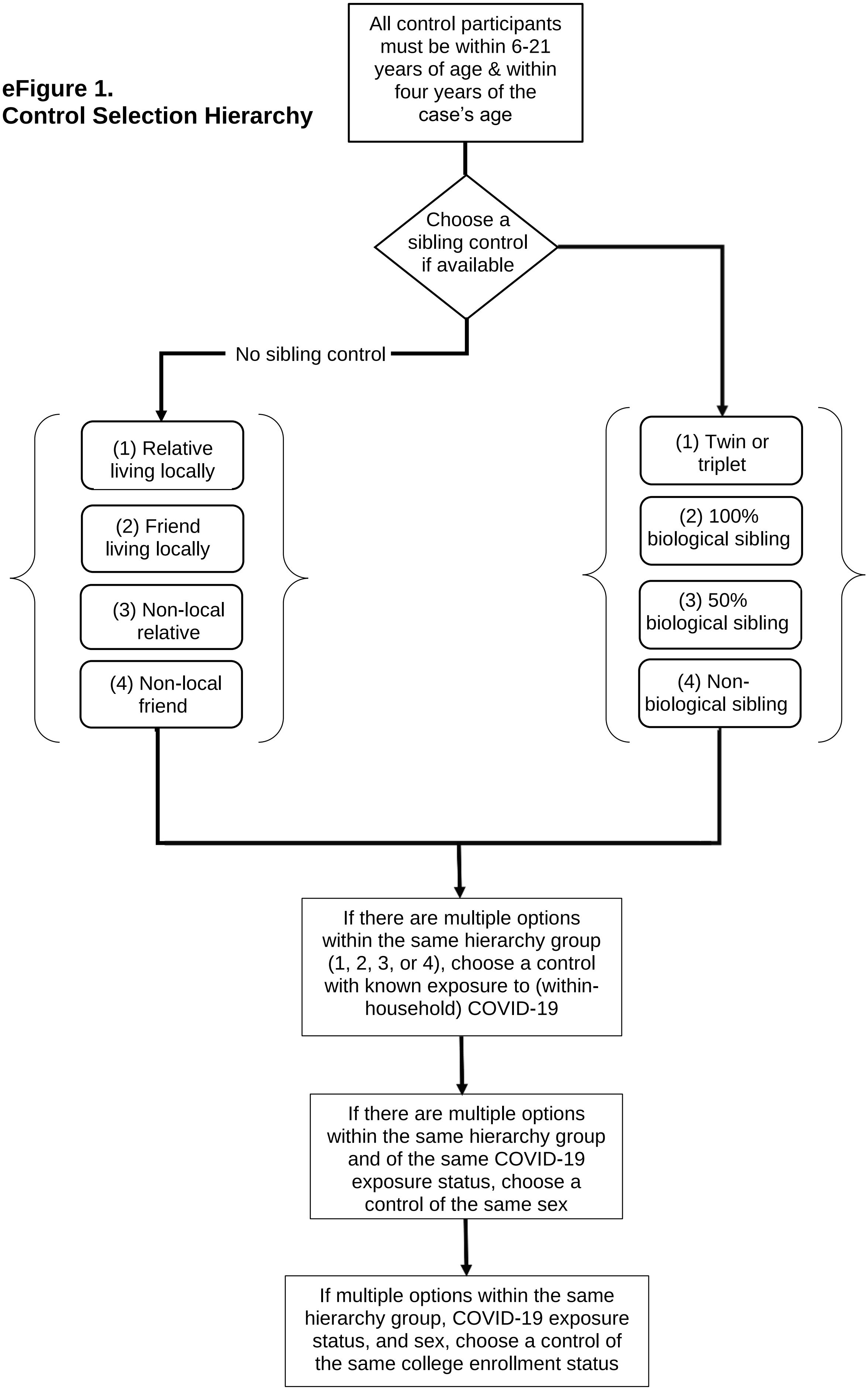

© 2023 Rollins CK et al. *JAMA Network Open.*

eFigure 2. Enrollment Flowchart

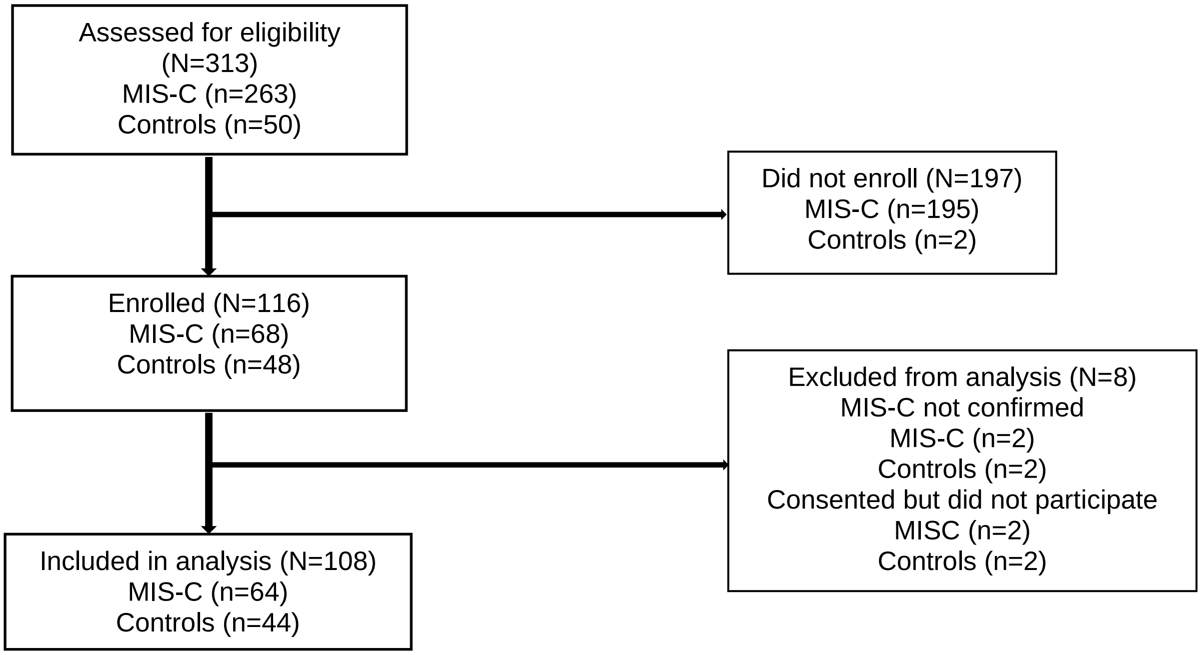

© 2023 Rollins CK et al. *JAMA Network Open.*

eTable 1. Abnormal Neurological Examination Findings by Group

Neurological Exam Component	MIS-C Participants, n/N (%) (N = 61)	Control Participants, n/N (%) (N = 43)
Cranial nerves		
Visual fields	0/61	0/43
Extraocular movements	0/61	0/43
Facial strength and symmetry	0/61	0/43
Hearing to finger rub	1/61 (2)	0/43
Motor[a]		
Axial tone	2/61 (3)	0/43
Upper extremity tone	3/50 (6)	0/34
Upper extremity strength[b]	2/61 (3)	1/43 (2)
Upper extremity symmetry of movement	0/60	0/43
Lower extremity tone	0/61	0/42
Lower extremity strength[b]	6/61 (10)	1/43 (2)
Lower extremity symmetry of movement	2/60 (3)	0/43 (1)
Finger Tapping	3/51 (6)	0/33
Finger Sequencing	3/51 (6)	1/33 (3)
Orbiting	0/60	0/42
Pronator Drift	1/61 (2)	0/42
Coordination		
Dysmetria	2/61 (3)	0/42
Heel-Knee-Shin	1/61 (2)	1/42 (2)
Gait		
Standard and Stress Gait Maneuvers[c]	3/61 (5)	1/42 (2)
Overall Impression Abnormal	15/61 (25)	3/43 (7)

[a]Motor exam conducted separately for left and right sides, combined here for ease of interpretation
[b]Confrontation testing performed by parent or other adult under guidance and direct observation of board-certified child neurologist who demonstrated how to isolate individual muscle groups and directly observed and solicited feedback to report findings
[c]Stress gait maneuvers included toe walk, heel walk, tandem gait, outside of feet, and inside of feet

© 2023 Rollins CK et al. *JAMA Network Open.*

eTable 2. Sensitivity Analysis Including Matched Pairs Only

Outcome[a]	MIS-C Participants mean (SD)	Control Participants, mean (SD)	Mean Difference MIS-C *vs* Controls (95% CI)[b]	Standardized Mean Difference MIS-C *vs* Controls (95% CI)[b,c]
Wechsler Intelligence Scales (WASI-II, WISC-V, WAIS-IV)				
IQ estimate	101.8 (13.2)	103.5 (14.2)	-1.7 (-6.1 to 2.7)	-0.11 (-0.41 to 0.18)
Working Memory Index	99.8 (15.3)	103.0 (15.5)	-3.2 (-8.8 to 2.4)	-0.21 (-0.59 to 0.16)
Processing Speed Index	95.5 (16.1)	96.8 (14.7)	-1.2 (-7.8 to 5.3)	-0.08 (-0.52 to 0.35)
NIH Cognition Toolbox				
List Sort Working Memory	98.7 (12.8)	103.1 (10.5)	-4.4 (-9.4 to 0.5)	-0.29 (-0.63 to 0.03)
Picture Sequence Memory	101.7 (15.8)	102.8 (18.0)	-1.1 (-7.0 to 4.8)	-0.07 (-0.47 to 0.32)
Delis-Kaplan Executive Function System (D-KEFS)				
Verbal Fluency Switching	10.7 (3.1)	10.8 (2.7)	0.0 (-1.4 to 1.3)	0.00 (-0.47 to 0.43)
Color-Word Interference Switching	9.1 (3.1)	9.6 (3.1)	-0.5 (-1.8 to 0.7)	-0.17 (-0.60 to 0.23)
BRIEF[d] Global Executive Composite	50.8 (9.5)	50.7 (10.7)	0.2 (-3.1 to 3.4)	0.02 (-0.31 to 0.34)
Behavior Assessment Scale for Children-3rd Edition (BASC-3)				
Internalizing Problems	50.3 (11.1)	47.6 (8.0)	2.7 (-0.8 to 6.2)	0.27 (-0.08 to 0.62)
Anxiety	48.3 (9.3)	49.5 (10.5)	-1.2 (-4.8 to 2.5)	-0.12 (-0.48 to 0.25)
Depression	49.8 (10.2)	47.9 (9.5)	1.9 (-1.6 to 5.4)	0.19 (-0.16 to 0.54)
Somatization	52.6 (12.8)	46.4 (7.0)	6.2 (2.0 to 10.1)	0.62 (0.20 to 1.01)
Externalizing Problems	48.7 (7.8)	47.0 (8.3)	1.6 (-1.2 to 4.5)	0.16 (-0.12 to 0.45)
Hyperactivity	48.9 (9.6)	47.1 (9.8)	1.8 (-2.2 to 5.7)	0.18 (-0.22 to 0.57)
Aggression	48.9 (7.2)	48.2 (7.0)	0.7 (-1.8 to 3.1)	0.07 (-0.18 to 0.31)
Conduct Problems	48.6 (6.7)	46.7 (7.7)	1.9 (-0.5 to 4.4)	0.19 (-0.05 to 0.44)
Behavioral Symptoms Index[e]	48.1 (8.2)	47.2 (10.0)	0.9 (-2.2 to 4.0)	0.09 (-0.22 to 0.40)
Atypicality	47.1 (7.4)	48.0 (11.0)	-0.9 (-4.6 to 2.8)	-0.09 (-0.46 to 0.28)
Withdrawal	48.5 (11.6)	48.1 (10.4)	0.3 (-4.0 to 4.7)	0.03 (-0.04 to 0.47)
Attention Problems	48.0 (11.5)	46.9 (9.5)	1.1 (-2.8 to 5.0)	0.11 (-0.28 to 0.50)
Adaptive Skills Composite	52.5 (10.1)	53.0 (11.2)	-0.5 (-3.6 to 2.5)	-0.05 (-0.36 to 0.25)
PedsQL Generic				
Self-report Total	83.0 (11.2)	85.2 (12.6)	-2.1 (-6.5 to 2.2)	-0.16 (-0.49 to 0.17)
Physical health	87.3 (14.2)	89.3 (12.2)	-1.9 (-6.6 to 2.7)	-0.14 (-0.48 to 0.19)
Psychosocial health	80.8 (11.5)	83.0 (14.8)	-2.3 (-7.1 to 2.6)	-0.16 (-0.48 to 0.18)
Parent-report Total	84.8 (11.1)	88.6 (13.2)	-3.8 (-8.0 to 0.5)	-0.24 (-0.50 to 0.03)
Physical health	91.6 (11.1)	90.9 (18.8)	0.8 (-5.5 to 7.0)	0.04 (-0.28 to 0.35)
Psychosocial health	81.2 (13.3)	87.4 (12.5)	-6.2 (-10.1 to -2.3)	-0.39 (-0.64 to -0.15)

© 2023 Rollins CK et al. *JAMA Network Open.*

Outcome[a]	MIS-C Participants mean (SD)	Control Participants, mean (SD)	Mean Difference MIS-C *vs* Controls (95% CI)[b]	Standardized Mean Difference MIS-C *vs* Controls (95% CI)[b,c]
PedsQL Multidimensional Fatigue				
Self-report Total	78.2 (16.0)	81.4 (14.3)	-3.3 (-7.8 to 1.3)	-0.25 (-0.59 to 0.10)
General fatigue	83.9 (15.8)	84.3 (18.2)	-0.5 (-7.2 to 6.3)	-0.03 (-0.48 to 0.42)
Sleep-rest fatigue	73.6 (22.3)	80.3 (17.0)	-6.6 (-12.9 to -0.4)	-0.35 (-0.69 to -0.02)
Cognitive fatigue	76.9 (20.3)	79.6 (18.0)	-2.7 (-9.3 to 3.9)	-0.15 (-0.53 to 0.22)
Parent-report Total	83.2 (17.8)	87.3 (17.1)	-4.1 (-10.5 to 2.4)	-0.36 (-0.92 to 0.21)
General fatigue	85.0 (19.0)	88.1 (18.8)	-3.0 (-10.3 to 4.2)	-0.23 (-0.77 to 0.32)
Sleep-rest fatigue	80.0 (22.8)	88.2 (16.4)	-8.2 (-16.3 to -0.1)	-0.55 (-1.11 to -0.01)
Cognitive fatigue	84.7 (19.7)	85.7 (24.3)	-1.0 (-9.5 to 7.4)	-0.07 (-0.63 to 0.49)
PROMIS Sleep Disturbance	52.4 (9.2)	50.8 (7.7)	1.6 (-2.1 to 5.3)	0.16 (-0.21 to 0.53)
PROMIS Sleep Impairment	47.6 (8.3)	47.5 (9.3)	0.1 (-2.7 to 2.9)	0.01 (-0.27 to 0.29)
Functional Disability Inventory	3.8 (5.9)	2.7 (4.0)	1.1 (-1.0 to 3.3)	0.28 (-0.25 to 0.83)

[a]Includes 44 Pairs; IQ Estimate (n = 41 pairs), Working Memory Index (n = 41), Processing Speed Index (n = 30; due to non-return of mailed booklets), List Sort Working Memory (n = 41), Picture Sequence Memory (n = 42), D-KEFS (n = 37; used only for age ≥8y), BRIEF (n = 43), BASC-3 (n = 39), PedsQL self- (n = 37) and parent-report (n = 37; due to survey non-completion), PROMIS (n = 39), and Functional Disability Inventory (n = 37)

[b]Adjusted for matched pairs

[c]Standardized mean difference is the effect size relative to 1 SD based on test parameters or normative data except for Functional Disability Inventory where sample SD was substituted given lack of normative data

[d]BRIEF = Behavior Rating Inventory of Executive Function, includes BRIEF-2 and BRIEF-A

[e]Includes depression, hyperactivity, aggression, atypicality, withdrawal, attention

© 2023 Rollins CK et al. *JAMA Network Open.*

eTable 3. Sensitivity Analysis Excluding Patients with ADHD (Unmedicated), Anxiety, and Autism

Outcome[a]	MIS-C Participants Score, mean (SD) (N = 53)	Control Participants Score, mean (SD) (N = 42)	Mean Difference MIS-C *vs* Controls (95% CI)[b]	Standardized Mean Difference MIS-C *vs* Controls (95% CI)[b,c]
Wechsler Intelligence Scales (WASI-II, WISC-V, WAIS-IV)				
IQ estimate	102.2 (13.8)	103.8 (14.3)	-0.8 (-5.2 to 3.7)	-0.05 (-0.37 to 0.25)
Working Memory Index	99.3 (15.2)	103.1 (15.8)	-3.5 (-9.3 to 2.4)	-0.23 (-0.62 to 0.16)
Processing Speed Index	98.3 (16.3)	96.8 (14.3)	0.9 (-5.4 to 7.2)	0.06 (-0.36 to 0.48)
NIH Cognition Toolbox				
List Sort Working Memory	95.9 (14.2)	102.5 (10.3)	-6.5 (-11.5 to -1.6)	-0.43 (-0.77 to -0.11)
Picture Sequence Memory	99.7 (14.4)	102.8 (18.2)	-2.5 (-8.4 to 3.4)	-0.17 (-0.56 to 0.23)
Delis-Kaplan Executive Function System (D-KEFS)				
Verbal Fluency Switching	10.6 (3.2)	10.7 (2.5)	-0.1 (-1.4 to 1.1)	-0.03 (-0.47 to 0.37)
Color-Word Interference Switching	8.9 (3.3)	9.8 (3.2)	-0.8 (-2.0 to 0.4)	-0.27 (-0.67 to 0.13)
BRIEF[d] Global Executive Composite	50.5 (9.5)	50.4 (10.8)	0.3 (-3.1 to 3.7)	0.03 (-0.31 to 0.37)
Behavior Assessment Scale for Children-3rd Edition (BASC-3)				
Internalizing Problems	51.7 (11.7)	47.8 (8.0)	3.8 (0.3 to 7.3)	0.38 (0.03 to 0.73)
Anxiety	49.4 (9.7)	49.4 (10.2)	-0.1 (-3.8 to 3.6)	-0.01 (-0.38 to 0.36)
Depression	50.6 (11.0)	47.8 (9.4)	2.8 (-0.8 to 6.4)	0.28 (-0.08 to 0.64)
Somatization	54.3 (14.2)	47.2 (7.7)	7.0 (2.8 to 11.4)	0.70 (0.28 to 1.14)
Externalizing Problems	48.0 (7.5)	47.2 (8.3)	0.9 (-2.0 to 3.7)	0.09 (-0.20 to 0.37)
Hyperactivity	48.3 (9.1)	47.8 (9.7)	0.4 (-3.2 to 4.0)	0.04 (-0.32 to 0.40)
Aggression	48.2 (7.3)	47.8 (6.6)	0.6 (-1.8 to 3.1)	0.06 (-0.18 to 0.31)
Conduct Problems	48.0 (6.5)	46.9 (7.9)	1.4 (-1.1 to 4.0)	0.14 (-0.11 to 0.40)
Behavioral Symptoms Index[e]	48.0 (8.2)	47.2 (10.2)	0.7 (-2.6 to 4.1)	0.07 (-0.26 to 0.41)
Atypicality	47.3 (7.2)	48.0 (11.1)	-0.9 (-4.7 to 2.9)	-0.09 (-0.47 to 0.29)
Withdrawal	48.9 (10.9)	47.4 (9.5)	1.8 (-2.1 to 5.6)	0.18 (-0.21 to 0.56)
Attention Problems	47.5 (11.1)	47.6 (10.4)	-0.4 (-3.9 to 3.2)	-0.04 (-0.39 to 0.32)
Adaptive Skills Composite	53.3 (10.0)	52.6 (11.4)	-0.0 (-3.2 to 3.2)	-0.00 (-0.32 to 0.32)
PedsQL Generic				
Self-report Total	81.8 (12.3)	86.1 (12.4)	-3.6 (-8.0 to 0.9)	-0.27 (-0.61 to 0.07)
Physical health	85.6 (15.9)	90.6 (10.6)	-4.4 (-9.1 to 0.3)	-0.32 (-0.66 to 0.02)
Psychosocial health	79.8 (12.5)	83.7 (14.7)	-3.2 (-8.2 to 1.9)	-0.22 (-0.56 to 0.13)
Parent-report Total	83.1 (12.0)	89.4 (12.9)	-5.5 (-9.9 to -1.2)	-0.35 (-0.62 to -0.08)
Physical health	88.6 (13.5)	92.1 (17.9)	-3.1 (-9.7 to 3.4)	-0.16 (-0.49 to 0.17)
Psychosocial health	80.2 (13.5)	87.9 (12.4)	-7.2 (-11.1 to -3.3)	-0.45 (-0.70 to -0.21)

© 2023 Rollins CK et al. *JAMA Network Open.*

**Outcome[a]**	**MIS-C Participants Score, mean (SD) (N = 53)**	**Control Participants Score, mean (SD) (N = 42)**	**Mean Difference MIS-C *vs* Controls (95% CI)[b]**	**Standardized Mean Difference MIS-C *vs* Controls (95% CI)[b,c]**
PedsQL Multidimensional Fatigue				
Self-report Total	77.8 (16.4)	82.4 (13.9)	-4.0 (-8.8 to 0.9)	-0.30 (-0.66 to 0.07)
General fatigue	81.9 (17.8)	87.0 (15.9)	-4.5 (-10.9 to 1.9)	-0.30 (-0.73 to 0.13)
Sleep-rest fatigue	74.7 (20.9)	80.9 (16.6)	-6.1 (-12.6 to 0.5)	-0.33 (-0.67 to 0.03)
Cognitive fatigue	76.9 (21.3)	79.4 (17.8)	-2.2 (-8.9 to 4.5)	-0.13 (-0.51 to 0.26)
Parent-report Total	81.0 (19.0)	88.4 (17.0)	-6.8 (-13.7 to 0.1)	-0.60 (-1.20 to 0.01)
General fatigue	81.9 (21.1)	90.5 (16.4)	-8.3 (-15.9 to -0.7)	-0.62 (-1.19 to -0.05)
Sleep-rest fatigue	79.7 (22.8)	89.5 (15.7)	-9.8 (-17.6 to -1.9)	-0.67 (-1.20 to -0.13)
Cognitive fatigue	81.3 (22.4)	85.3 (24.5)	-2.2 (-10.7 to 6.2)	-0.15 (-0.71 to 0.41)
PROMIS Sleep Disturbance	52.7 (9.4)	50.3 (7.4)	2.4 (-1.0 to 5.8)	0.24 (-0.10 to 0.58)
PROMIS Sleep Impairment	48.0 (9.5)	47.0 (9.2)	0.6 (-2.4 to 3.6)	0.06 (-0.24 to 0.36)
Functional Disability Inventory	3.6 (5.5)	2.6 (3.9)	1.0 (-0.9 to 2.9)	0.25 (-0.23 to 0.74)

[a]IQ Estimate (MIS-C n = 52, control n = 40), Working Memory Index (n = 53, 39), Processing Speed Index (n = 38, 33; due to non-return of mailed booklets), List Sort Working Memory (n = 51, 39), Picture Sequence Memory (n = 53, 40), D-KEFS (n = 43/44, 36/38; given to ages ≥8y), BRIEF (n = 52, 41), BASC-3 (n = 48, 38), PedsQL self- (n = 46, 37) and parent-report (n = 49, 36; due to survey non-completion), PROMIS (n = 50, 37), and Functional Disability Inventory (n = 46, 38)
[b]Adjusted for matched pairs
[c]Standardized mean difference is the effect size relative to 1 SD based on test parameters or normative data except for Functional Disability Inventory where the control sample SD was substituted due to lack of well-established normative data
[d]Behavior Rating Inventory of Executive Function, includes BRIEF-2 and BRIEF-A
[e]Includes depression, hyperactivity, aggression, atypicality, withdrawal, attention

© 2023 Rollins CK et al. *JAMA Network Open.*

eTable 4. Associations Between Selected Demographic and Hospitalization-Related Medical Variables[a] and Neuropsychological Outcome Measures[b] Among MIS-C Participants

	Predictor Variable[a]			
	SVI	LVEF[d]	ICU/Non-ICU	Neurologic Presentation
**Outcome[b]**	**Beta Coefficient[c] (95% CI) n**	**Beta Coefficient[c] (95% CI) n**	**Mean Difference (95% CI) n**	**Mean Difference (95% CI) n**
List Sort Working Memory	-24.6 (-38.3 to -10.8) n = 60	-3.8 (-7.8 to 0.2) n = 60	-3.9 (-10.9 to 3.2) n = 62	-6.3 (-15.7 to 3.2) n = 60
Verbal Fluency Switching	-2.3 (-5.2 to 0.5) n = 50	-0.9 (-1.8 to 0.1) n = 51	-0.8 (-2.5 to 0.9) n = 52	0.0 (-1.9 to 2.0) n = 50
Color-Word Interference Switching	-1.7 (-5.7 to 2.2) n = 50	-1.4 (-2.3 to -0.4) n = 51	-1.9 (-3.6 to -0.3) n = 52	-2.1 (-4.9 to 0.8) n = 50
BASC Internalizing Problems	3.7 (-11.6 to 19.0) n = 56	-2.5 (-5.4 to 0.3) n = 56	-6.3 (-13.3 to 0.8) n = 58	5.5 (-3.7 to 14.7) n = 56
BASC Depression	4.2 (-9.9 to 18.3) n = 56	-2.2 (-4.9 to 0.5) n = 56	-9.9 (-16.2 to -3.5) n = 58	2.2 (-7.0 to 11.5) n = 56
BASC Somatization	4.1 (-13.3 to 21.4) n = 56	-2.0 (-5.6 to 1.5) n = 56	0.0 (-8.0 to 8.0) n = 58	12.1 (2.4 to 21.8) n = 56
Behavioral Symptoms Index	8.6 (-5.4 to 22.6) n = 56	-1.7 (-4.1 to 0.7) n = 56	-7.5 (-13.7 to -1.3) n = 58	-4.1 (-10.3 to 2.1) n = 56
PedsQL Generic (Self- Report)	-4.2 (-18.5 to 10.0) n = 55	3.6 (0.5 to 6.6) n = 54	6.5 (-0.3 to 13.2) n = 56	-1.3 (-10.2 to 7.6) n = 54
Neurological Exam (Abnormal), Odds Ratio (95% CI)	1.3 (0.1 to 19.7) n = 59	1.5 (0.8 to 3.0) n = 59	2.2 (0.6 to 7.4) n = 61	2.2 (0.5 to 8.9) n = 59

[a] Predictor variables assessed: Social Vulnerability Index (SVI) , primary caregiver education, length of stay (continuous), length of stay dichotomized (>7 days vs not), need for intensive care, intensive care unit length of stay, severe hemodynamic compromise (shock requiring vasopressors, need for cardiopulmonary resuscitation, or extracorporeal membranous oxygenation), highest CRP, steroid exposure, lowest left ventricular ejection fraction (LVEF) category, neurological symptoms at presentation, neurological examination at follow-up evaluation

[b] Outcome variables assessed: NIH Cognition Toolbox List Sort Working Memory, D-KEFS verbal fluency switching, D-KEFS color-word interference switching, BASC-3 internalizing problems, BASC-3 depression, BASC-3 somatization, BASC-3 behavioral symptoms index, Peds-QL Generic (self-report), neurological examination at follow-up

[c] Beta coefficients from regressions are not standardized

[d] Lowest left ventricular ejection fraction coded as 0 = normal, 1 = mild (45-54%), 2 = moderate (35-44%), or 3 = severe (<35%) depression

© 2023 Rollins CK et al. *JAMA Network Open.*
