# Supplementary material for: Neurological and Psychological Sequelae Associated With Multisystem Inflammatory Syndrome in Children
Source: JAMA Netw Open. 2023 Jul 19;6(7):e2324369. doi: 10.1001/jamanetworkopen.2023.24369 (PMC10357334; doi:10.1001/jamanetworkopen.2023.24369)
Supplement: Supplement 2. — Nonauthor Collaborators [file jamanetwopen-e2324369-s002.pdf]

Supplemental Online Content: Nonauthor Collaborators
*First name, last name, and suffix (if applicable) are required and will appear in PubMed.

*Group Name(s): Overcoming COVID-19 Investigators							
***First Name and Middle Initial(s)**	***Last Name**	***Suffix (eg, Jr, III)**	Academic Degrees	Institution	Location (city, state/province, country)	Role or Contribution, eg, chair, principal investigator	Group (if more than 1 Group listed in the byline) and/or Subgroup (eg, Steering Committee)
Heather	Kelly		RN	Children's of Alabama	Birmingham, AL	Nurse Coordinator	
Meghan	Murdock		RN	Children's of Alabama	Birmingham, AL	Nurse Coordinator	
Lora	Martin		NP	Children's Hospital of Mississippi	Jackson, MS	Nurse Practitioner	
Lacy	Malloch		BA	Children's Hospital of Mississippi	Jackson, MS	Project Manager	
Ashley	Stanley-Copeland		MD	Children's Hospital of Mississippi	Jackson, MS	Investigator	
Jeanie	Craft		BA	Children's Hospital of Mississippi	Jackson, MS	Coordinator	
