# Supplementary material for: Neurological and Psychological Sequelae Associated With Multisystem Inflammatory Syndrome in Children
Source: JAMA Netw Open. 2023 Jul 19;6(7):e2324369. doi: 10.1001/jamanetworkopen.2023.24369 (PMC10357334; doi:10.1001/jamanetworkopen.2023.24369)
Supplement: Supplement 3. — Data Sharing Statement [file jamanetwopen-e2324369-s003.pdf]

Data Sharing Statement

Rollins. Neurological and Psychological Sequelae Associated With Multisystem Inflammatory Syndrome in Children. *JAMA Netw Open*. Published July 19, 2023.
doi:10.1001/jamanetworkopen.2023.24369

Data
Data available: Yes
Data types: Deidentified participant data
How to access data: Upon request: caitlin.rollins@childrens.harvard.edu
When available: beginning date: 01-01-2024

Supporting Documents
Document types: None

Additional Information
Who can access the data: Researchers whose proposed use of the data has been approved.
Types of analyses: For a specified purpose.
Mechanisms of data availability: After approval of a proposal.
